# Seroprevalence of *Toxoplasma gondii* infection among babies in Jeddah Province, Saudi Arabia: a retrospective study

**DOI:** 10.11604/pamj.2022.42.72.32877

**Published:** 2022-05-26

**Authors:** Raafat Abdel Moneim Hassanein, Adel Galal El-Shemi, Mohammed Othman Alkurbi, Ameer Ahmed Alahmadi, Waslallah Saad Almatery

**Affiliations:** 1 Department of Laboratory Medicine, Faculty of Applied Medical Sciences, Umm Al-Qura University, Mecca, Saudi Arabia,; 2 Department of Zoonoses, Faculty of Veterinary Medicine, Assiut University, Assiut, Egypt,; 3 King Fahad Hospital, Jeddah, Saudi Arabia

**Keywords:** *Toxoplasma gondii*, Saudi babies, seroprevalence

## Abstract

**Introduction:**

toxoplasmosis is an opportunistic protozoan disease caused by Toxoplasma gondii (T. gondii) infection. It affects all human ages, including children, and can pose serious health problems, particularly in developing countries. Nevertheless, the epidemiological status of neonatal and childhood toxoplasmosis remains largely unknown in Saudi Arabia. The present study aimed to determine the seroprevalence of T. gondii infection among Saudi babies residing in Jeddah Region of Saudi Arabia.

**Methods:**

this hospital-based retrospective study was conducted between January 2019 and March 2021 at three governmental hospitals in Jeddah Region: King Fahad, King Abdulaziz, and East Jeddah Hospital. It included 502 babies (269 boys and 233 girls; 0-4 years old), who were screened by enzyme linked immunosorbent assay (ELISA) for the detection of anti-T. gondii immunoglobulin G (IgG) and immunoglobulin M (IgM) antibodies in their serum.

**Results:**

among the 502 studied babies, the overall seropositivity rate of T. gondii infection was 18.53% (93/502) subscribed as 90 babies (17.9%) with IgG seropositive and 3 babies (0.60%) with IgM seropositivity. The all IgG seropositive babies were IgM seronegative and vice versa. Additionally, the highest proportion of IgG seropositivity was detected in 0-6 month old babies (7.17%); followed by 5.38% and 4.98% in 7-12 and 13-18 months old babies, respectively, while the 3 babies with IgM seropositivity were 13-18 months old.

**Conclusion:**

the present findings highlighted the seroprevalence situation of toxoplasma infection among babies in some Saudi communities and raise the importance to increase the screening programs and preventative implements against toxoplasmosis in Saudi Arabia.

## Introduction

Toxoplasmosis is a globally widespread protozoan zoonotic disease caused by infection with *T. gondii*. The disease represents a major public health threat, and can affect all human ages, particularly in developing countries [1,2]. Approximately one-third of the world human population has chronic toxoplasmosis, and 30% to 50% of its overall endemicity is localized in the Middle East countries [1-3]. The life cycle of *T. gondii* includes a sexual stage arising in the primary host (i.e, domestic and wild cats), and asexual stage that occurs in the secondary hosts of humans and warm-blooded animals. Each cat can excrete over 20 million oocysts of *T. gondii* within 4 -13 days post-infection; and these oocysts survive in the environment for long period [4,5]. Ingestion of oocysts shed by cats into the environment-and ingestion of raw meat, non-boiled milk, and undercooked food products of infected farm animals, as well drinking of water containing *T. gondii* cysts, are the main horizontal routes of transmission of human toxoplasmosis [5-7]. Transmission of *T. gondii* through blood transfusion and organ transfer should not be neglected [8].

At the same time, infection with *T. gondii* during pregnancy can induce spontaneous abortion and it may be vertically transmitted to fetus; resulting in congenital toxoplasmosis [9]. The incidence and clinical severity of congenital toxoplasmosis is influenced by the stage of pregnancy, the strength of mother and fetus immune response, and the fetal environment characteristics [1,10]. Variable clinical signs have been observed in infants born with congenital toxoplasmosis including ocular damage (retinochoroiditis and blindness), hydrocephalus, macro or microcephalus, cerebral calcifications, delayed neuropsychomotor development and mental retardation [10,11]. Moreover, the European multicenter studies have previously demonstrated that treatment of toxoplasmosis in infected pregnant women does not significantly prevent the risk of its vertical transmission to fetuses [12,13]. Most importantly, in children born with asymptomatic congenital toxoplasmosis, a reactivation or relapse form of the disease, with life-threatening sequences, has been significantly reported during their neonatal to childhood life, or even in their puberty life [14-17].

The less effectiveness of health education practices in preventing the incidence of *T. gondii* infection [18,19], besides the unavailability of vaccine, may increase the impacts of toxoplasmosis on all human ages. Thus, establishment of maternal and neonatal screening programs for toxoplasmosis is an essential demand to improve the child health, particularly when coupled with an effective early treatment [19-21]. Toward this goal, the present study was conducted to highlight the seroprevalence proportion of *T. gondii* infection among a population of Saudi babies living in Jeddah Province in the Western Region of Saudi Arabia.

## Methods

**Study design, setting and participants:** the present study is a hospital-based retrospective study that was carried out to evaluate the seroprevalence rate of *T. gondii* infection among a population of Saudi babies living in Jeddah Province in the Western Region of Saudi Arabia. In this study, the medical records of a total of 502 Saudi babies (0-4 years old; 269 boys and 233 girls), who were subjected to healthcare check-up and clinical monitoring for infectious diseases at three governmental hospitals in Jeddah Region: King Fahad, King Abdulaziz, and East Jeddah Hospital, between January 2019 and March 2021, were analyzed. Ethical approval for this study was obtained from the Institutional Review Board of the Ministry of Health, Directory of Health Affairs-Jeddah, Kingdom of Saudi Arabia (ethical approval number: 1461). The minimal study sample size was estimated according to the previously published worldwide reports. Jeddah is the largest city in the Western Region of Saudi Arabia with high population densities and a unique situation on the Red Sea coast, and the above three hospitals were selected in order to maximize the variety of our study sample as they are the main clinical, teaching and training healthcare medical services in Jeddah.

**Data sources/measurement:** the hospital-based serological screening for *T. gondii* infection was carried out by ELISA-based estimation of the specific anti-*T. gondii* IgG and IgM antibodies in serum samples using commercial ENZYWELL TOXOPLASMA IgG and IgM Kit (Siemens Healthcare Diagnostics Products GmbH, Marburg, Germany) [22,23]. According the manufacturer´s instructions, samples with absorbance higher than the cut-off value (i.e. >1.3 for IgG and >1.2 for IgM) were considered as seropositive, while those with absorbance of <0.7 for IgG and <0.8 for IgM were considered as seronegative. Samples with borderline values (0.7-1.3 for IgG and 0.8-1.2 for IgM) were considered as doubtful (equivocal) results and repeated 2-3 weeks later to be verified as positive or negative [22,23].

**Statistical analysis:** data entry and analysis were done using SPSS software package version 20.0 (SPSS Inc. Chicago, Illinois, USA). The Chi-square (χ^2^) test and student “t” test or Mann-Whitney test were used for the categorical data and continuous variables as appropriate. A P-value of <0.05 was considered statistically significant.

## Results

A total of 93 (18.53%) babies, among the studied 502 babies, were detected to have *T. gondii* infection sub-divided as follow: 17.93% (90/502) were identified as seropositive for anti-*T. gondii* IgG antibodies (i.e. IgG+), and 0.60% (3/502) were identified as seropositive for anti-*T. gondii* IgM antibodies (i.e. IgM+) (Table 1). Additionally, the all babies presented IgG seropositivity were IgM seronegative and vice versa; babies who had IgM seropositivity were IgG seronegative (Table 1). According to the babies´ sex (Table 2), out of the 90 babies who had IgG seropositivity, 49 (54.44%) were boys and 41 (45.56%) were girls, while the 3 babies who had IgM seropositivity were 2 boys and one girl (Table 2).

**Table 1:** the overall seroprevalence of anti-*T. gondii* IgG and IgM antibodies in the studied babies

	IgG+ no (%)	IgM+ no (%)	IgG+/ IgM- no (%)	IgG-/ IgM+ no (%)	IgG+/ IgM+ no (%)
Presented	90/502 (17.93)	3/502 (0.60)	90/502 (17.93)	3/502 (0.60)	0/502 (0.0)
Not-presented	412/502 (82.1)*	499/502 (99.4)*	412/502 (82.1)*	499/502 (99.4)*	502/502 (100.0)
Total	502/502 (100.0)	502/502 (100.0)	502/502 (100.0)	502/502 (100.0)	502/502 (100.0)

IgG+: IgG seropositivity; IgG-: IgG seronegativity; IgM+: IgM seropositivity; IgM-: IgM seronegativity; *P <0.05

**Table 2:** prevalence of anti-*T. gondii* IgG and IgM antibodies seropositivity according to the sex of the studied babies

Sex	N	IgG seropositivity no (%)	IgM seropositivity no (%)
Boys	269	49/269 (18.22%)* [49/502 (9.76%)]*	2/269 (0.74%) [2/502(0.4%)]
Girls	233	41/233 (17.60%)* [41/502(8.17%)]*	1/233 (0.43%) [1/502(0.2%)]
Total	502	90/502 (17.93%)*	3/502 (0.6%)

* P <0.05 IgG+ vs IgM+

Next, to try to figure out possible role of baby´s age on the seroprevalence rate of toxoplasmosis (Table 3), we found that the highest proportion of IgG seropositivity (7.17%) was in 0 to 6 months old babies; followed by a proportion of 5.38%, 4.98% and 0.4% in babies with age of 7-12 months, 13-18 months, and 19-24 months old, respectively. No IgG seropositivity was detected in other age sets. On the other hand, the all 3 babies presented IgM seropositivity (3/502; 0.6%) were with age of 13-18 months old (Table 3). Lastly, no-specific toxoplasmosis clinical sequelae were detected in the medical records of all studied babies.

**Table 3:** prevalence of anti-*T. gondii* IgG and IgM antibodies seropositivity according to the ages of the studied babies

Age group (months)	IgG seropositivity no (%)	IgM seropositivity no (%)
0-6	36/502 (7.17)*	0/502 (0.0)
7-12	27/502 (5.38)*	0/502 (0.0)
13-18	25/502 (4.98)*	3/502 (0.6)*
19-24	2/502 (0.40)*	0/502 (0.0)
26-30	0/502 (0.0)	0/502 (0.0)
31-36	0/502 (0.0)	0/502 (0.0)
37-42	0/502 (0.0)	0/502 (0.0%)
43-48	0/502 (0.0)	0/502 (0.0)
Total	90/502 (17.93)*	3/502 (0.6)

* P <0.05

## Discussion

Infection with *T. gondii* is one of the most prevalent parasitic zoonotic diseases affecting all human ages worldwide. However, there are scarce evidence concerning the status of relevant of gestational (maternal)/congenital, postnatal and childhood toxoplasmosis in Saudi Arabia [24]. The present hospital-based retrospective study was therefore, undertaken to determine the seroprevalence rates of *T. gondii* infection in a sample population of male and female Saudi national babies residing in Jeddah Province in the Western Region of Saudi Arabia. The study was conducted between January 2019 and March 2021 at three governmental hospitals in Jeddah Region. The age of the study babies ranged from 0 to 4 years old, and the sero-screening procedures of *T. gondii* infection was based on detection of the specific anti-*T. gondii* IgM and IgG antibodies by ELISA [22-25]. The results showed that among the studied 502 babies, 93 (18.53%) babies were detected to have *T. gondii* infection in form of 17.93% and 0.60% positive to anti-*T. gondii* IgG and IgM antibodies, respectively. Additionally, all babies who had IgG+ were IgM-, and vice versa; all babies with IgM seropositivity were IgG seronegative. It is generally recognized that the detection of anti-*T. gondii* IgM antibodies denotes an acute phase of the disease, while the detection of anti-*T. gondii* IgG antibodies indicates a state of chronic infection [22-25]. However, anti-toxoplasma IgG antibodies may be also transmitted passively from the mothers to their newborns and are not endogenously created antibodies [26].

In harmony with our findings, Al-Yami *et al*. [24] have recently detected the presence of 21.0% and 0.8% seropositivity for anti-*T. gondii* IgG or IgM antibodies, respectively, in 500 paired samples from maternal and newborn (cord blood samples) during child delivery in the Eastern Province of Saudi Arabia [24]. Additionally, data of previous reports concerning the prevalence of toxoplasmosis among the “adult individuals” from different regions in Saudi Arabia have been reviewed and revealed that the seroprevalence of anti-toxoplasma IgG (+) antibodies ranged from 9.13% in the Hail Region to 39.43% in the Eastern Region, while the seroprevalence of its specific IgM (+) antibodies ranged from 0.44% in the northern region to 17.7% in the eastern region of Saudi Arabia [27]. Most interestingly, our current findings are also in consistency with those of some worldwide studies. For instance, the weighed prevalence of IgM+/symptomatic *T. gondii* infection has been estimated as 0.396% to 0.835% among the Mexican newborns and children during 1954 to 2009 [28]. Furthermore, Danish Congenital Toxoplasmosis Study Group (Denmark) has previously disclosed that a neonatal serological screening program for 12 months post-delivery and based on detection of IgM antibodies alone can effectively recognize 70%-80% of cases of congenital toxoplasmosis [29].

Changes in environmental and climate conditions, differences in host susceptibility; hygiene facilities and other socioeconomic conditions, and complexity pattern of *T. gondii* transmission and variability in its diagnostic methods, inevitably lead to distinct variations in the seoprevalence rates of positive *T. gondii* infection at the global, regional, and country levels, and even between the different regions of a country [30,31]. In a constant line, though its influence is not determined yet in children, several reports have suggested the central role of age in the variation of toxoplasmosis prevalence among adult individuals [1,2,27]. We herein observed that the highest proportion of IgG seropositivity was in 0 to 6 months old babies; followed by those with age of 7-12 months and 13-18 months and the all three cases of IgM seropositivity were reported in babies with age of 13-18 months old. Our findings are nearly in matching with those reported previously in Poland [32]. The explanatory reasons beyond these findings are not well known; however, it may attribute to one or more of the following probabilities: passive transmission of anti-*T. gondii* IgG antibodies from chronically infected mothers [26]; vertical transmission of *T. gondii* during pregnancy/labor [33]; or an exposure to sources of *T. gondii* infection during/post-labor process. At that regard, a previous report from Brazil indicated that from 487 infected pregnant mothers, 7 newborns (1.42%) were confirmed to have vertically transmitted acute *T. gondii* infection [34]. These collective data argue for deeper investigations to explore the potential risk factors that threat the prevalence of toxoplasmosis during the neonatal and childhood life.

**Limitations:** in addition to the study's main findings, important limitations were inevitably identified, that should be addressed in the future. First, in IgM (+) babies there are difficulties in defining the source of these anti-*T. gondii* IgM antibodies if they were a result of a vertically transmitted, neonatal, postnatal acquired *T. gondii* infection, or due to a reactivation of a latent congenital infection [15]. Thus, it was had to add a second level of assessments such as IgG avidity test, immunoblotting test, or polymerase chain reaction (PCR) detection of *T. gondii* (DNA) to identify the time/source of the infection [35]. Second, there is also a conflict in defining the source of anti-*T. gondii* IgG antibodies in their seropositive babies if they were secondary to congenital or postnatal *T. gondii* infection, or they were transmitted passively from the immunized mothers to their newborns and did not represent endogenously created antibodies [26]. Third, there is a notable lack in the information related to variables that might had an influence on the seroprevalence of *T. gondii* infection among the studied babies; such as immune status of the babies, maternal toxoplasmosis screening data, and type of labor (normal or cesarean). Fourth, the present data were drawn from limited samples of Saudi babies resident in the Western Region of Saudi Arabia that in turn may not necessarily have represented the overall national seroprevalence rate of toxoplasmosis. Coherently, further large scale and multi-centers prospective screening and follow-up studies are essentially required; in which a second level of the above mentioned examinations will be added. The same can be also said for pregnant women to prevent the risk of vertically transmitted congenital toxoplasmosis.

## Conclusion

Findings of the present study showed that there was 17.93% and 0.6% seropositivity prevalence for anti-*T. gondii* IgG and IgM antibodies, respectively, among the 502 studied babies, who were resident in Jeddah Province of Saudi Arabia between January 2019 and March 2021. These findings can in turn raise a dire need to increase the screening implements and improve the preventative programs against *T. gondii* infection in the Kingdom of Saudi Arabia.

### What is known about this topic

- There is scarce evidence in the literature related to the incidence of congenital, neonatal and childhood toxoplasmosis in Saudi Arabia;
- The neonatal and childhood screening program for toxoplasma disease in Saudi Arabia remains challenging.

### What this study adds

- The present findings of the current study provide an insight into the prevalence of toxoplasmosis among babies in the Kingdom of Saudi Arabia;
- The present findings can also serve as a starting point for increasing the neonatal and childhood screening policies and preventative programs against T. gondii infection in the Kingdom of Saudi Arabia.
